# Special Issue: Molecular and Cellular Mechanisms of Cardiovascular Diseases

**DOI:** 10.3390/biomedicines14010047

**Published:** 2025-12-25

**Authors:** Antonella Galeone, Giacomina Brunetti

**Affiliations:** 1 Department of Surgery, Dentistry, Pediatrics and Gynecology, Division of Cardiac Surgery, University of Verona, 37126 Verona, Italy; 2 Department of Biosciences, Biotechnologies and Environment, University of Bari Aldo Moro, 70125 Bari, Italy; giacomina.brunetti@uniba.it

Cardiovascular diseases are a leading cause of death worldwide [1]. Recent large-scale genomic studies have demonstrated the significant influence of genetic variation on the risk of cardiovascular diseases [2], with some being related to aging, genomic instability, telomere shortening, and the loss of proteostasis [3]. Various studies are ongoing to better understand the molecular and cellular mechanisms that lead to cardiovascular disease, highlighting the involvement of inflammation, senescence, mitochondrial dysfunction, and metabolic disturbances [2,3,4]. Here, we report the latest findings derived from publications in this Special Issue.

Balmos et al. (contribution 1) deepened the pathogenesis of atherosclerosis, a progressive disease arising from inflammatory arterial wall disorder, endothelial dysfunction, and atheromatous plaque formation. Consequently, carotid artery stenosis develops, resulting in ischemic injury and atherothrombotic stroke. The study involved sixty-seven patients showing perilesional inflammatory cell infiltrate. Around the atheroma lipid core, CD31+ endothelial cells together with CD68+, iNOS2+ and Arg1+ macrophages were found through digital morphometry, and the respective levels correlated with ulceration, thrombosis, plaque hemorrhage, calcification patterns, and neo-vessel development as determinants of instability. Interestingly, patients showing intraplaque hemorrhaging showed higher CD68+ macrophage infiltration, whereas in 12 other cases where iNOS2 was much more highly expressed compared with Arg1, the manifestation of atherothrombotic events was significantly more recurrent. CD31 levels are positively related to atherothrombosis. Thus, these authors concluded that atherothrombosis is associated with the presence of neovessels and pro-inflammatory iNOS2+ macrophages, consequently meaning that controlling macrophage polarization will represent an additional therapeutic approach to preventing plaque destabilization.

Baloglu (contribution 2) focused on the Na,K-ATPase (NKA) pump’s role in promoting optimal activity of the heart. NKA activity diminished in necropsy samples from heart failure and ischemic heart disease and in experimental models. The cellular response to hypoxia is modulated by hypoxia-induced transcription factors (HIF); thus, the authors evaluated HIF’s involvement in the modulation of the levels and intracellular dynamics of the α2-isoform of NKA (α2-NKA). HIF-1α and HIF-2α levels were strongly reduced in H9c2 cardiomyocytes by adenoviral infection, where cells were kept in 1% O_2_ for 24 h. The authors showed that hypoxia augmented the levels of α2-NKA mRNA by 5-fold with respect to normoxic cells in an HIF-2α-sensitive manner. α2-NKA plasma membrane levels were augmented in hypoxia by 2-fold and were completely inhibited by HIF-2α silencing. α2-NKA intracellular expression was not modulated.

Canale et al. (contribution 3) reported on telomere dysfunction, which is involved in vascular aging, with shorter leucocyte telomeres being related to an augmented risk of atherosclerosis, heart failure, and myocardial infarction. An additional pathophysiological mechanism supporting the relationship between atherosclerosis development and telomere shortening focuses on the clonal hematopoiesis of indeterminate potential (CHIP), representing a new and independent risk factor in atherosclerotic cardiovascular diseases. As telomere attrition has a central involvement in sustaining vascular senescence, the comprehension of telomere biology is fundamental to preventing the harmful consequences of vascular aging, together with its cardiovascular disease-related manifestations. Promising evidence shows that a class of long noncoding RNAs transcribed at telomeres, identified as TERRA or “TElomeric Repeat-containing RNA”, dynamically contributes to the mechanisms modulating telomere preservation and chromosome end protection. The role of TERRA in vascular biology is unknown. In their review, the authors discuss the recent knowledge presented on TERRA, together with its functions in telomere biology. Furthermore, the authors describe the relationship between TERRA dysregulation and disease, suggesting its potential role in new therapeutic approaches.

Torarek et al. (contribution 4) reviewed the pathogenetic mechanisms of cardiovascular disease and diabetes, highlighting the shared pathways of these entities and the prominent role of the microbiota. Metabolic anomalies, such as hyperglycemia, hyperlipidemia, inflammation, and insulin resistance, lead to molecular events in the cardiovascular system, affecting both vessels and the myocardium. The development of diabetic heart disease is caused by inflammation, increases in oxidative stress, increases in the levels of advanced glycation end products (AGEs), the expression of mRNAs, the occurrence of epigenetic factors, vascular remodeling, imbalances of cell death, the dysregulation of mitochondria, endoplasmic reticulum stress, lipotoxicity, cardiac hypertrophy, and cardiac fibrosis. Insulin, biguanides, thiazolidinediones, gliptins, glucagon-like peptide 1 analogs, and glifozins are all used to treat diabetes. Among these, glifozins inhibit sodium–glucose transporters 2 (SGLT-2) and are currently indicated in the treatment of both diabetes and cardiovascular disease, due to their beneficial effects on the cardiovascular system. Besides their ability to reduce glucose levels, SGLT-2 inhibitors present additional benefits such as lowering blood pressure and reducing the risk of hospitalization due to heart failure. The mechanisms by which SGLT-2 inhibitors reduce the risk of cardiovascular disease include natriuresis with a decreased plasma volume and a subsequent increase in hematocrit levels, improved vascular function, reductions in adipose-tissue-mediated inflammation, the lower production of pro-inflammatory cytokines, the conversion of ketone bodies into cardiac and renal metabolic substrates, reductions in oxidative stress, reductions in serum uric acid levels, and the inhibition of AGEs signaling.

Liantonio et al. (contribution 5) analyzed the main scientific advancements in the identification of biomarkers for the diagnosis, risk stratification, pathophysiology and treatment of Brugada syndrome, an inherited cardiac channelopathy that may cause sudden cardiac death. To date, variants in more than 25 different genes have been linked to Brugada syndrome, including genes encoding ion channel subunits and regulatory proteins. The SCN5A gene, encoding for the alpha subunit of the cardiac voltage-gated sodium channel Nav1.5, accounts for about 20–30% of Brugada syndrome cases and it is the only one considered clinically valid. More than 300 different variants in SCN5A have been identified, and these are linked to various clinical phenotypes and have also been associated with dilated cardiomyopathy. However, the molecular mechanisms remain unknown in 70–85% of clinically confirmed cases. In fact, many patients show a non-Mendelian inheritance and present rare variants in non-coding regions or unknown genes, and even the presence of copy number variations in genes affecting the onset of Brugada syndrome is emerging as a possible pathogenetic mechanism.

Brunetti et al. (contribution 6) reported the current knowledge on the role of the ST2/IL-33 pathway in adult and pediatric heart disease and transplantation. ST2 is a member of the interleukin 1 receptor family, with soluble sST2 and transmembrane ST2L isoforms, and its ligand is IL-33. The ST2/IL-33 pathway is primarily involved in inflammatory and autoimmune diseases, but it also has a major role in the cardiovascular system and diseases such as ischemic heart disease, heart failure, heart valve disease, pulmonary arterial hypertension, and vascular disease, as well as heart transplantation. sST2 levels increase in the serum of patients after AMI and correlate negatively with the left ventricular ejection fraction, and predict mortality and heart failure in these patients. Serum sST2 levels are also significantly higher in patients with severe chronic HF and correlate with BNP levels; additionally, a change in sST2 levels over time is an independent predictor of subsequent mortality or the need for heart transplantation in these patients. Elevated sST2 levels in heart recipients are associated with cellular rejection and predict long-term mortality following a heart transplant. In particular, serum sST2 levels rise significantly in the context of acute rejection after a heart transplant, show a significant linear association with the severity of acute rejection, and significantly decline after successful rejection therapy.

## References

[1] GBD 2023 Cardiovascular Disease Collaborators (2025). Global, Regional, and National Burden of Cardiovascular Diseases and Risk Factors in 204 Countries and Territories, 1990–2023. J. Am. Coll. Cardiol..

[2] Pauklin S., Qiao J., Cai L., Chang M., Wang C., Zhao R., Song S., Tan N., He P., Jiang L. (2025). Shared genetic architecture contributes to risk of major cardiovascular diseases. Nat. Commun..

[3] Zhang S.Y., Yang Y.H., Wen R., Yang N., Feng S.S., Zhang T.N. (2025). Cellular and molecular mechanisms underlying cardiovascular aging. Cell Mol. Biol. Lett..

[4] Li Y., Du J., Deng S., Liu B., Jing X., Yan Y., Liu Y., Wang J., Zhou X., She Q. (2024). The molecular mechanisms of cardiac development and related diseases. Sig. Transduct. Target. Ther..

